# Distribution of polyphenolic and sugar compounds in different buckwheat plant parts†

**DOI:** 10.1039/d1ra04250e

**Published:** 2021-07-28

**Authors:** Milica Nešović, Uroš Gašić, Tomislav Tosti, Nikola Horvacki, Nebojša Nedić, Milica Sredojević, Stevan Blagojević, Ljubiša Ignjatović, Živoslav Tešić

**Affiliations:** Institute of General and Physical Chemistry Studentski trg 12-16 11158 Belgrade Serbia; Department of Plant Physiology, Institute for Biological Research “Siniša Stanković”, National Institute of Republic of Serbia, University of Belgrade Bulevar Despota Stefana 142 11060 Belgrade Serbia uros.gasic.@ibiss.bg.ac.rs; University of Belgrade – Faculty of Chemistry Studentski trg 12-16 11158 Belgrade Serbia ztesic@chem.bg.ac.rs; Innovation Center, University of Belgrade – Faculty of Chemistry Studentski trg 12-16 11158 Belgrade Serbia; Faculty of Agriculture, Institute for Zootehnics, University of Belgrade Nemanjina 6 11080 Belgrade – Zemun Serbia; University of Belgrade – Faculty of Physical Chemistry Studentski trg 12-16 11158 Belgrade Serbia

## Abstract

The aim of this study was to provide information on the phenolic and sugar profiles of different parts of the buckwheat plant, which can define that buckwheat is a functional food, with a high nutritional value and very useful for human health. Therefore, the extracts of buckwheat leaf, stem, and flower, as well as buckwheat grain were analysed for the content of polyphenol and antioxidant tests. The identification of a notable number of phenolic compounds and quantification of sugars in different parts of buckwheat indicates that buckwheat is a highly valuable plant. A total of 60 phenolic compounds were identified (18 cinnamic acid derivatives, 14 flavonols, 13 flavan-3-ols (including proanthocyanidins), 10 hydroxybenzoic acid derivatives, and 5 flavones) using ultra-high-performance liquid chromatography (UHPLC), coupled with a hybrid mass spectrometer which combines the Linear Trap Quadrupole (LTQ) and OrbiTrap mass analyzer. The highest number of phenolic compounds was found in the analysed buckwheat flower sample, and then in the leaf, followed by the grain and the stem. In addition, the sugar profile of buckwheat leaf, stem, flower and grain, as well as the buckwheat pollen and the nectar was analysed. Hence, 16 sugars and 5 sugar alcohols were detected by the high-performance anion exchange chromatography (HPAEC) with a pulsed amperometric detector (PAD). Sucrose was found in a significant amount with the highest content in buckwheat leaf. Trisaccharides had similar accumulation in the sample extracts, while disaccharides dominated in buckwheat leaf, followed by nectar and pollen. The sugar alcohols showed the highest content in buckwheat grain, where erythritol was predominant. The obtained results show that buckwheat is very rich in phenolic compounds and sugars. In addition to grain, the other parts of the buckwheat plant can be used as a very good source of different classes of phenolic compounds. This study provides useful information on the distribution of phytochemicals in different parts of the buckwheat plant, which contribute to the maintaining of the status of buckwheat as a functional food.

## Abstract 2

The aim of this study was to provide information on the phenolic and sugar profiles of different parts of the buckwheat plant, which can define that buckwheat is a functional food, with a high nutritional value and very useful for human health.

## Graphical abstract

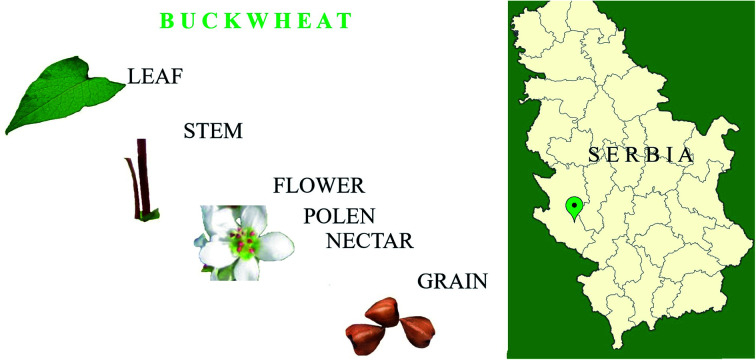

## Introduction

Buckwheat (*Fagopyrum esculentum* Moench) belongs to the pseudo-cereals and as such it is recognized as a food with high nutritional and nutraceutical value.^1^ It does not contain gluten, which is one of its health-supporting properties that further contribute to its use in a diet.^2^ Due to its use, similar food preparation and conditions of growing, buckwheat is very close to the cereals. Nevertheless, buckwheat, as well as other pseudo-cereals, is closer to the fruits and vegetables due to the content of compositions of polysaccharides.^3^ Buckwheat has a high content of vitamins thiamin and riboflavin, well-balanced proteins, phenolic compounds, soluble carbohydrates and dietary fibre.^3^

In general, plants show insensitivity of sugar translocation to the stress conditions, which allows them to use food reserves where they are needed.^4^ However, the impact on sugars' synthesis and their ratio through the plant is dependent on the stress conditions and the cultivar which was analysed by many authors.^5–9^ The influence of stress was also noted for buckwheat sugar content,^10^ as well as for rutin content.^11^

Carbohydrates are the primary products of photosynthesis, and they provide nutritional properties to the buckwheat. By the analysis of the buckwheat grain, it was found that the most representative sugars were glucose, fructose, arabinose and xylose, as well as sucrose and maltose.^1^ As primary metabolites, the plant polysaccharides are characteristic precursors for the polyphenol synthesis^12^ and they differ from bioactive secondary metabolites due to their hydrophilicity and biocompatibility,^13^ while their biological activities significantly overlap.^3^

Buckwheat represents the highest source of polyphenols among other pseudo-cereals, as was stated by Martínez-Villaluenga *et al.*,^1^ and when compared to cereals such as oat and barley, buckwheat possesses higher antioxidant activity.^14,15^ The phenolic compounds, such as monomeric flavanol 3-ols and B type procyanidins, are important for buckwheat, as some of them were not reported, or they were found in a lower amount in cereals,^16,17^ which are more used in the diet. On the contrary, it was reported that there are lower values of dehydrodiferulic acid and dehydrotriferulic acid in buckwheat than in cereals.^3^ According to Zielińska *et al.*^18^ the nutraceutical properties of buckwheat have been mainly attributed to the presence of several flavonoids. The prominent compound in different parts of buckwheat was found to be rutin,^18,19^ the levels of which reduce during seed ripening.^20^ The significant observation was that buckwheat is the only pseudo-cereal that possesses rutin flavonoid.^21^

The most common uses of buckwheat in the diet are, for example, buckwheat stem as a salad, leaves and flowers as a tea, grain as a porridge *etc.*, whereas all its parts could be used. Furthermore, buckwheat products such as flour and honey are widely used. The use of buckwheat grains for the filling of pillows is also known. As a functional food, buckwheat is recognized as a valuable plant for research. There are published papers based on analysing buckwheat as a whole plant,^16,22^ its seed,^10,11,22–24^ root,^19,25^ stem,^18,19,22,25^ leaf,^11,18,19,22,26–28^ flower,^18,19,22,25,29^ grain^17,18^ and hull,^14,24^ as well as its products such as flour,^14,30^ bran,^24^ honey^31,32^ and tea.^22^ Most published studies are based on the examination of phenolic compounds,^11,14,16,18,19,22–27,29–32^ antioxidant activity,^14,18,23,27–31^ as well as some other parameters such as primary metabolites and genus as a response to salt stress,^10^ α-tocopherol^29^ and squalene content,^19^ content of fagopyrin,^22^ dietary fibres,^25^ changes during the fermentation,^28^ and sugar content.^31^

Buckwheat grain has a tetrahedral form, which sets it apart from the grains of other crops. The most common buckwheat products for the diet are flour and porridge, obtained from the hulled grains or groats. The low glycemic index of buckwheat groats^33^ and high content of total phenolic in the groats^24^ contribute to its nutritional and health-supporting properties. A successive blooming of buckwheat in a period of 2–3 months produce a vast number of flowers grouped in inflorescences. Therefore, there is an abundance of nectar and pollen that attract a large number of insects.^1^ It was showed that the buckwheat pollen contained the highest fructose content, as well as the fructose/glucose ratio relative to other pollen samples.^34^ Buckwheat flowers showed a higher content of rutin in comparison with buckwheat leaf.^22^

Buckwheat plant (*Fagopyrum esculentum* Moench) variety ‘Novosadska’ showed the highest content of polyphenols and antioxidant capacity with respect to twelve other buckwheat varieties.^23^ Also, a significant yield of production of buckwheat variety ‘Novosadska’ in Serbia was reported.^2^ Furthermore, a notable number of the identified polyphenols in ‘Novosadska’ buckwheat nectar showed a good correlation with a phenolic profile of buckwheat honey, as well as with buckwheat pollen.^31^

In order to expand the investigation related to ‘Novosadska’ buckwheat, the different parts of this plant were analysed. Therefore, the phenolic profile and the antioxidant capacity of ‘Novosadska’ buckwheat leaf, stem, flower and grain were analysed, and compared with those in the corresponding buckwheat pollen, nectar and honey, previously published.^31^ In addition, the sugar profile in the buckwheat extracts of leaf, stem, flower, grain, as well as in the pollen and nectar, was analysed. Furthermore, the idea was to study the transport of the sugars and polyphenols from the beginning of photosynthesis to the other parts of buckwheat which could be used for human nutrition. Additionally, by analysing buckwheat nectar and pollen, which bees use as food, the idea was to compare the phytochemical content of pollen and nectar with buckwheat plant as well as buckwheat honey previously published. By this comparison, it could be possible to characterize the botanical origin of buckwheat honey, which is known to have great pollen variability. Moreover, the focus of this study was to increase the attention to different parts of buckwheat as a good source of bioactive compounds, with the aim to highlight the buckwheat as a functional food.

## Experimental

### Samples

**Fig. 1:**
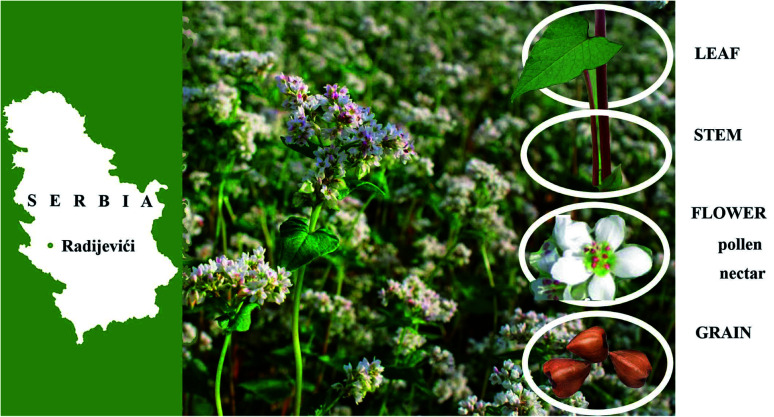
Buckwheat samples (leaf, stem, flower, grain, pollen, nectar) collected in Serbia, Nova Varoš, Radijevići (43°23′31′′ N; 19°52′20′′ E).

All samples were taken from the buckwheat plant (*Fagopyrum esculentum* Moench) species ‘Novosadska’ cultivated in a small locality in Western Serbia, in a village Radijevići (43°23′31′′ N, 19°52′20′′ E) (Fig. 1). Samples were harvested at the flowering season of buckwheat during the full nectar secretion, in July 2017. Buckwheat plant organs such as leaf, stem, flower, and grain (Fig. 1), were separated. At the same time, buckwheat nectar and pollen samples were directly collected from buckwheat flowers, by the procedure reported by Nešović *et al.*^31^ In order to avoid the contamination of flower sample, the flowers from which pollen and nectar were taken were not used in further analysis.

### Reagents, standards and materials

The chemicals for extraction were of the analytical grade. The reagents for the determination of total phenolic content and the relative scavenging activity (Folin–Ciocalteu's reagent, gallic acid, 2,2-diphenyl-1-picrylhydrazyl (DPPH), Trolox standard), the reagents for the mobile phases for sugar analysis (sodium hydroxide, sodium acetate), and chemicals for phenolic analysis (hydrochloric acid, acetonitrile, formic acid) were purchased from Sigma Aldrich (Steinheim, Germany). Phenolic standards (gallic acid, protocatechuic acid, 3-*O*-caffeoylquinic acid, 5-*O*-caffeoylquinic acid, *p*-hydroxybenzoic acid, caffeic acid, *p*-coumaric acid, ferulic acid, catechin, epicatechin, luteolin 6-*C*-glucoside, myricetin, luteolin 8-*C*-glucoside, quercetin 3-*O*-(6′′-rhamnosyl)-glucoside, apigenin 8-*C*-glucoside, quercetin 3-*O*-glucoside, (epi)catechin gallate, quercetin 3-*O*-rhamnoside, luteolin 7-*O*-glucoside, luteolin, quercetin, kaempferol, kaempferide) were also purchased from Sigma Aldrich (Steinheim, Germany).

The sugar standards for glucose, fructose, xylose, arabinose, sucrose, maltose, trehalose, and maltotriose were supplied from Tokyo Chemical Industry, Europe (Zwijndrecht, Belgium), isomaltose, melibiose, gentiobiose, raffinose, panose were purchased from Tokyo Chemical Industry, TCI (Tokyo, Japan), while the rest of the sugar standards (turanose and sugar alcohols) were obtained from Sigma-Aldrich (Steinheim, Germany).

Ultrapure water 18 MΩ cm (0.055 μS cm^−1^) was produced by TKA MicroPure water purification system (Thermo Fisher TKA). The filter paper (Whatman no 1) was supplied by Merck (Darmstadt, Germany) and the syringe filters (13 mm, PTFE membrane 0.45 μm) were supplied by Supelco (Bellefonte, PA). For the solid-phase extraction (SPE) SPE cartridges were used (Strata C18-E, 500 mg 3 mL^−1^), obtained from Phenomenex (Torrance, CA).

### Preparation of the sample extracts

Buckwheat samples (leaf, stem, flower, grain) were separately dried, then crushed, and well ground. The dehydration was performed in the dark place at the room temperature for 15 days. The samples were put in plastic vials and stored in the darkness until they were analysed. The sample extracts were prepare in triplicate and the results were presented as g kg^−1^ of dry weight (dw).

The extraction of different parts of buckwheat (leaf, stem, flower and grain) for both chromatography (UHLC OrbiTrap MS) and spectrophotometric analyses was performed by a modified method reported by Dziedzic *et al.*^25^ The amount of each dried leaf, stem, flower and grain sample (∼0.1 g) was extracted by 10 mL of aqueous methanol solution MeOH/H_2_O (70/30) acidified with 0.1% HCl. After one hour of homogenizing in an ultrasonic bath, the samples were centrifuged (15 min; 14 152 g) and filtered. After the residue was removed, the extract solutions of samples were diluted to the concentration of ∼0.1 g L^−1^ and used for subsequent analyses.

The solid-phase extraction of buckwheat pollen and nectar sample were prepared by the method described by Gašić *et al.*^35^ the polyphenols from buckwheat pollen and nectar samples were extracted using aqueous solutions, acidified with 0.1% HCl to pH 2. After conditioning the SPE cartridges with 3 mL of acetonitrile and 9 mL of ultrapure water, the sample extract solutions were passed through the cartridges. The first eluate was washed with ultrapure water and collected for the sugar analysis. The cartridges with absorbed phenolic compounds were dried by the nitrogen gas, after which the polyphenols were eluated with acetonitrile. The extraction of sugars from the rest parts of buckwheat (leaf, stem, flower and grain) was performed by dissolving the samples (∼0.1 g) in 10 mL of ultrapure water, after which they were diluted for 50 times.^8^

### Determination of sugar profile

The determination of sugar content, as well as the preparation of sugar standard solutions, was performed under conditions which were described by Fotirić-Akšić *et al.*^8^ High-performance anion-exchange chromatograph with pulsed amperometric detection (HPAEC/PAD) (Dionex ICS 3000, Sunnyvale, CA, USA) was used, which consisted of the quaternary gradient pump, analytical CarboPac PA 100 column (4 × 250 mm), and 3 mobile phases (600 mM sodium hydroxide, 500 mM sodium acetate and ultrapure water). The validation parameters for sugar quantification (Table S1†), as well as the chromatogram of quantified sugar compounds (Fig. S1†) is presented in ESI.†

### Statistical analysis

Tukey's test was used to evaluate the differences (*p* ≤ 0.05) between the mean values of sugars (NCSS software package). PLS_Tool Box software package for MATLAB (Version 7.12.0) was used to perform Principal Component Analysis (PCA). Prior to PCA, the data were group-scaled, a singular value decomposition algorithm (SVD) and a 0.95 confidence level for Q and Hotelling T2 limits for outliers were set.

### Determination of antioxidant tests

Determination of total phenolic content (TPC) was based on the spectrophotometric method with Folin Ciocaleu's reagent, according to the procedure described by Dziadek *et al.*,^27^ with minor modifications. The sample extract solutions (0.1 mL) were mixed with 0.5 mL of ultrapure water and 2.5 mL of Folin Ciocaleu's reagent. After shaking the mixture and leaving it for 5 minutes, it was added 2 mL of sodium carbonate.

The determination of radical scavenging activity (RSA) was based on the spectrophotometric method with DPPH reagent, according to the procedure described by Inglett *et al.*^30^ Briefly, 0.1 mL of sample extract solution was mixed with 4 mL of DPPH methanol solution.

For the measurement of the absorbance for both TPC and RSA it was used UV/Vis spectrophotometer (GBC UV/Visible Cintra 6, Australia) at the wavelength of 765 nm and 517 nm, sequently.

### Determination of phenolic profile

The solutions of phenolic available standards were prepared in methanol in the concentration range of 0.025 to 1.000 mg L^−1^, as was described by Gašić *et al.*^35^

The phenolic compounds were identified using liquid chromatography system. Ultra high-performance liquid chromatograph was connected to the linear ion trap and with mass spectrometer (UHPLC-LTQ OrbiTrap MS; Thermo Fisher Scientific, Bremen, Germany). The following conditions were previously described by Gašić *et al.*^35^

Syncronis C18 column (100 × 2.1 mm, 1.7 μm particle size) from Thermo Fisher Scientific was used for peak separation. Flow rate was set at 0.300 mL min^−1^ and the mobile phase was consisted of (A) water + 0.1% formic acid and (B) acetonitrile + 0.1% formic acid. The injection volumes were 5 μL and linear gradient programs were as follows: 0.0–1.0 min 5% (B), 1.0–16.0 min from 5% to 95% (B), 16.0–16.1 min from 95% to 5% (B), and 5% (B) for 4 min.

The MS data were acquired in negative ionization mode in the full-range acquisition covering 100–1000 *m*/*z*. Resolution was set to 30 000 for full scan analysis. The data-dependent MS2 events were always performed on the most intense ions detected in the full scan MS.

This system provides MS^*n*^ the fragmentation of deprotonated molecules, which was compared with those of the available standards and the published data. Verifying the presence of phenolic compounds was based on their fragmentation pathways, the retention time and the comparison between accurate mass and the calculated mass.

## Results and discussion

### Sugar profile

Table 1 presents the obtained data for the quantified sugars in the analysed buckwheat sample extracts. Additionally, each mean value was followed by different letter, which represents result of Tukey's test (Table 1).

**Table 1:** Content of sugars (g kg^−1^ of dry weight) in buckwheat (*Fagopyrum esculentum*) samples: leaf, stem, flower, grain, pollen and nectar^a^

Parameter	Buckwheat samples					
	Leaf	Stem	Flower	Grain	Pollen	Nectar
Glucose	255.54^b^	251.33^b^	293.52^a^	264.73^b^	58.86^d^	132.06^c^
Fructose	274.87^d^	424.70^a^	359.85^c^	394.01^b^	168.17^f^	204.09^e^
Xylose	0.05^a^	0.01^e^	0.04^b^	0.02^d^	0.01^e^	0.03^c^
Arabinose	0.07^a^	0.01^d^	0.04^b^	0.01^d,e^	0.03^c^	0.01^e^
Rhamnose	0.02^c^	0.01^d^	0.03^b^	0.01^e^	0.03^a^	0.01^f^
Sucrose	270.46^a^	54.51^d^	36.99^e^	48.13^d^	135.07^b^	106.14^c^
Maltose	4.99^b^	3.24^c^	4.95^b^	2.85^d^	13.44^a^	1.54^e^
Isomaltose	0.79^a^	0.01^d^	0.02^d^	0.01^e^	0.05^c^	0.05^b^
Trehalose	0.03^c^	0.02^c^	1.12^b^	0.03^c^	0.03^c^	3.97^a^
Turanose	0.01^b^	ND^g^	0.01^b^	ND^g^	0.02^a^	0.02^a^
Melibiose	0.27^a^	0.01^e^	0.04^b,c^	0.03^d^	0.04^c,d^	0.05^b^
Gentiobiose	0.01^d^	0.01^d^	0.03^c^	0.01^e^	0.05^b^	0.06^a^
Melezitose	0.02^c^	0.02^c^	0.03^b^	0.01^d^	0.12^a^	0.12^a^
Raffinose	0.05^b^	0.02^c^	0.02^c^	0.01^d^	0.07^a^	0.07^a^
Maltotriose	0.30^a^	0.05^c^	0.24^b^	0.02^e^	0.03^d,e^	0.04^c,d^
Panose	0.03^c^	0.12^a^	0.02^c,d^	0.01^d^	0.09^b^	0.09^b^
Erythritol	2.51^d^	4.73^c^	7.15^b^	18.61^a^	0.36^e^	0.43^e^
Glycerol	0.58^c,d^	1.55^b^	0.37^d^	2.41^a^	0.60^c^	ND^g^
Sorbitol	0.24^b^	0.32^a^	0.03^e^	0.06^d^	0.07^d^	0.17^c^
Galactitol	ND^g^	0.39^b^	ND^g^	0.01^c^	1.83^a^	0.01^c^
Mannitol	0.37^c^	1.18^b^	1.63^a^	0.01^e^	ND^g^	0.19^d^
Fructose/glucose (F/G) ratio	1.08^d^	1.69^b^	1.23^d^	1.49^c^	2.86^a^	1.55^b,c^
Sum of monosaccharides	530.55^b^	675.97^a^	653.48^a^	658.78^a^	227.10^d^	336.19^c^
Sum of disaccharides	276.56^a^	57.81^d^	43.15^e^	51.06^d^	148.70^b^	111.84^c^
Sum of trisaccharides	0.40^a^	0.20^c^	0.31^b^	0.05^d^	0.31^b^	0.32^b^
Sum of sugar alcohols	3.70^d^	8.16^c^	9.19^b^	21.11^a^	2.86^e^	0.80^f^
Total sugars	811.21^a^	742.14^b^	706.13^c^	731.00^b^	378.97^e^	449.15^d^

a ND – not detected. Different letters in the same row denote a significant difference among varieties according to Tukey's test, *p* < 0.05.

In the analysed buckwheat leaf, stem, flower, grain a high content of sugars (706.13–811.21 g kg^−1^ dw) was found, while pollen and nectar taken from the buckwheat flowers showed about two-fold smaller values (378.97 and 449.15 g kg^−1^ dw, respectively). It was detected 21 sugars: five monosaccharides, seven disaccharides, four trisaccharides, and five sugar alcohols (Table 1). Monosaccharides were classified as dominant in the analysed buckwheat sample extracts, starting from stem, and followed by grain, flower and leaf (Table 1). This was in the line with the observations of other authors,^6^ who reported the accumulation of low sugars in the stem before anthesis.

Glucose and fructose were the most abundant monosaccharides. The ratio of glucose and fructose goes in favour of fructose in all analysed samples. It was stated that fructose has a lower glycemic index, opposite to glucose, as well as higher fructose/glucose (F/G) ratio produces a lower glycemic index.^34^ The low glycemic index, which has already been reported for buckwheat groats,^33^ attracts attention in terms of their use as functional foods. In the view of plant parts of buckwheat (leaf, stem, flower, grain), the stem possessed the highest fructose concentration (424.70 g kg^−1^ dw), as well as the highest F/G ratio (1.69) (Table 1). According to the fructose content and F/G ratio, the stem buckwheat extract was followed by the grain, the flower and then the leaf extract (Table 1). The relative high F/G ratio in buckwheat grain (1.49) could suggest that the process of grain ripening was not completed. Considering that sucrose has been reducing to glucose and fructose during the process of ripening, the obtained F/G ratio should be closer to 1. As the samples at the time of buckwheat flowering were collected, which is a successive process, it could be expected that the buckwheat grains were not fully formed. The obtained high values of F/G ratio for the nectar (1.55) and the pollen sample (2.86) indicate that these samples also have a low glycemic index.^34^ This was expected, as it was already noted that honey, obtained by processing pollen and nectar, possess low glycemic index, even lower than glucose.^34^

Sucrose was the next dominant sugar in the analysed samples. Its stands to be the major disaccharide, with abundant content in the buckwheat leaf sample (270.46 g kg^−1^ dw) (Table 1). As the leaf is the place where photosynthesis is mainly performed, it could lead to dominant occurrences of some sugars. Sucrose metabolism has a main contribution to polyphenols formations.^12^ Also, as a transport sugar, it is mostly responsible for translocation into the phloem.^4^ Despite, it has not been excluded that the other parts of buckwheat contribute to the process of photosynthesis. Nevertheless, it was found that the high content of soluble sugars (glucose, fructose, and sucrose) decreased the transport of photosynthesis products from leaf to the other buckwheat organs,^9^ which is in the line with our obtained results for sucrose. When the content of monosaccharides is concerned, the smallest amount in the buckwheat leaf was found (Table 1). The reason for this could be a higher accumulation of rutin, which has been reported to have an effect on the decreasing of glucose content.^36^ Moreover, the higher production of rutin in buckwheat leaf was already published by many authors.^11,18,19^ A high content of sucrose in the leaf sample extract, as well as the low F/G ratio (Table 1), could also be a consequence of some kind of plant stress.^9^ Considering that the high content of sucrose in the buckwheat leaf extract sample (Table 1) takes the amount of ∼30% of total sugars in this extract, it was comparable to the quantified sucrose content in the leaf sample of cucumber plant under the cold stress.^7^ Furthermore, Taiz *et al.*^4^ reported that an increase in sucrose content was associated with an increase in cold tolerance. Since the buckwheat variety ‘Novosadska’ was successfully grown at higher altitudes,^2^ low temperature conditions could be expected.

Without sucrose, the values of the sum of disaccharides show more similarity through the buckwheat sample extracts (Table 1). In addition to sucrose, the next dominant content of all sugars was found to be maltose, which follows the reported results for pseudo-cereals.^1^ Observing the content of other disaccharides, the leaf possessed higher content of maltose, isomaltose, melibiose, while trehalose and gentiobiose had higher contents of the buckwheat flower extract sample (Table 1).

The sum of disaccharides was dominant in the buckwheat leaf extract, followed by the pollen and the nectar. In the buckwheat pollen extract sample the highest amount of maltose (13.44 g kg^−1^ dw) was found (Table 1). It was also emphasized that maltose, arabinosis, galactitol, and melibiose could be influenced by plants stress.^8^

After the disaccharides, the sugar alcohols possessed the next dominant portion of carbohydrates. The sums of sugar alcohols were in the range from 0.2% (for buckwheat nectar extract) to 2.9% (for buckwheat grain extract) of the sum of total sugars in analysed samples. The concentration of erythritol, as the next dominant compound in sample extracts, may be arranged as follows: analysed the buckwheat grain, flower, stem, leaf, nectar and pollen (Table 1). Erythritol has less sweetness than sucrose and does not affect the blood sugar levels. Furthermore, it is a valuable sugar for functional beverages.^13^ In addition to erythritol, glycerol also contributes to the noticeably higher content of the sum of sugar alcohols in the grain than in the other analysed samples. Sorbitol is a photosynthate, as well as a translocated sugar alcohol. The obtained results of the higher accumulation of sorbitol in leaf than in grain, agree with the other reported results.^5^

Contrary to the highest content of the sum of sugar alcohols in the buckwheat grain sample extract, a content of the sum of trisaccharides was more than three times lower than in the other analysed samples (Table 1). The quantified trisaccharides had the poorest contribution to the content of all sugars (Table 1). Their amount in the buckwheat leaf extract was higher than in stem, flower and grain extract sample. Trisaccharide raffinose was reported as the phloem transport oligosaccharide in many plant families.^37^ Its accumulation in the sample extract was in range of 0.01–0.07 g kg^−1^ dw. Similar to the already published influence of plant stress on many sugars,^5,8–10^ the content of raffinose could be affected by abiotic stress.^7^

The flowering process, which was the period of sampling, as well as some other processes in the plant physiology, represents a stress for the plant. The process of preparing the plant for the fertilization or nectar secretion, as well as for the production of grain, require the additional energy and has caused many reaction processes. Observing the obtained sugar content for the buckwheat flower sample extract, as with the effects that stress causes on the plant, a certain correlation can be created. In addition to stress influence on raffinose,^7^ the salt stress^10^ could cause higher fructose and glucose content, while the low K levels^5^ could make the lowest content of sorbitol (Table 1). Contrary to the flower, the stem extract possessed the highest concentration of sorbitol as well as fructose content (Table 1). Within this, the stem should be considered as an organ with the dominant xylem transport. Depending on the adaptation of the plant on water potential, the synthesis of commonly accumulated compatible components (which also include sucrose, sorbitol) can be distributed differently in plant organs.^4^

Comparing the obtained results of total sugar content for buckwheat nectar and pollen extract, higher values were found in nectar. However, in the pollen extract was found higher content of maltose and sucrose, as well as the sum of sugar alcohols, due to the high presence of galactitol. It could be noted that the difference based on the sugar content, between buckwheat nectar and pollen, was less obvious than the previously reported differences in the presence of polyphenols in them.^31^ Among seven different types of pollen, Kalaycıoglu *et al.*^34^ reported the highest fructose content and F/G ratio for the buckwheat pollen. However, we obtained higher F/G ration for buckwheat pollen (2.86 in opposite to theirs value of 2.54 (ref. 34)).

The sugar content in buckwheat draw attention due to their comparison to other cereals, as was stated by Huda *et al.*^20^ Considering the results of sugar content for the analysed buckwheat sample extracts, it could be noted that each analysed part of buckwheat possessed a valuable amount of carbohydrates. However, it should be emphasized that the sugar accumulation depends on plant adjusting to stress, as well as on plant phenology,^6^ which determines the available place for the sugar reservoir. Furthermore, the significant quantified content of sugars certainly affects the content of phenolic compounds, which may be present in forms conjugated to sugars. Additionally, each analysed buckwheat sample extract showed the potential to improve the positive properties of buckwheat as a functional food, which was already reported for pseudo-cereals grain.^3^

### Statistical analysis

In order to indicate the possible variability in sugar profiles among the examined buckwheat (*Fagopyrum esculentum*) extract samples, PCA was performed. PCA included data of 16 sugars and five sugar alcohols. The obtained four-component model explained 91.52% of the total variance.

Score plots revealed some clustering of the investigated samples (Fig. S2A†), and the most influential variables were identified using the loading plots (Fig. S2B†). Leaf and flower separated from the other samples along the PC2 axis due to higher contents of maltotriose and xylose. Moreover, leaf sample stood out with significantly higher amounts of some sugars (sucrose, isomaltose, melibiose, and arabinose) when compared to the rest of the samples. Observing the PC1 axis, it could be noticed that nectar and pollen formed a cluster on the opposite part of score plot from the leaf, flower, grain, and stem. High contents of turanose, gentiobiose, melezitose, and raffinose, and low contents of glucose, fructose and erythritol were characteristics of nectar and pollen.

With PCA analysis performed on sugar profile data, it was confirmed previously noted variance between the different parts of buckwheat. In order to obtained more obvious differentiation, it is better to combine several groups of analysed parameters. However, applied PCA on one group of parameters mainly gave a good classification results of different parts of the plant, as was already noted in the literature.^38^

### Antioxidant tests

The highest obtained values of TPC and RSA were found for the buckwheat flower extract sample, following with the leaf extract. Superiority in the number of phenolic compounds (discussed in further section) for the buckwheat flower and the leaf extract indicate higher values of antioxidant activity, TPC and RSA values (Table 1).

The obtained results of TPC for the buckwheat the flower, the leaf, the grain and the stem extract were 68.37, 52.63, 12.99 and 6.85 g GAE kg^−1^ dw, respectively. The obtained TPC values were in the same order of magnitude with the published results for the buckwheat flower,^29^ leaf and seed,^15^ hull and bran.^24^ Lower TPC values than we obtained were reported for the buckwheat hull^14^ and flour,^14,17,30^ while higher TPC levels were confirmed for *Fagopyrum tataricum*.^39^ It is interesting to mention that the ripening process influence the content of total flavonoids in buckwheat leaves and flowers by increasing their levels from the early flowering to the period of full flowering and the seed formation, while their in the stem decrease.^18^ This was in accordance with our obtained results, as TPC values for the leaf and the flower extracts were more than 7 and 9 times, respectively, higher than in the stem extract sample. The similar comparisons between plant parts and stem have been noted for other species.^12^

The obtained results of RSA for the buckwheat flower, leaf, stem and grain extract were 447.96, 374.58, 319.77 and 317.12 mmol TE kg^−1^ dw, respectively. Our obtained following the order for RSA values (flower > leaf > grain) was also in accordance with the observations by other authors.^15,18^ It could be noted that lower values were reported by Zielinska *et al.*^18^ who analysed buckwheat parts in different period (early flowering, as well as flowering and seed formation). Analysing their results, a high differences between periods of sampling could be noted. However, a long-time of buckwheat flowering, as well as its overlapping with the grain formations should be taken into consideration. Hence, it was difficult to identify the exact moment of harvesting, which will be in accordance with the observations of other authors. The same order of magnitude for the activity that was obtained was noted for buckwheat flower,^29^ flour^30^ and *Fagopyrum tataricum* buckwheat.^39^

The obtained result for Pearson's coefficient (0.95) pointed out a good linear correlation between DPPH scavenging activity and the spectrophotometrically determined phenolic content for the analysed buckwheat sample extracts. This was in accordance with the results obtained by other authors.^28^ However, considering the point of view of the number of identified polyphenols (discussed in further section), the obtained RSA values shown to be less changeable in comparison with TPC values.

### Phenolic profile

Using UHPLC OrbiTrap MS technique, 59 phenolic compounds (28 phenolic acids and their derivatives and 31 flavonoids and their derivatives) were detected (Table 2). Chromatograms of some identified phenolic compounds in buckwheat extracts are presented on Fig. S2.† The compounds were confirmed using the standards and the previously published MS data.^27,31,35,40–51^

**Table 2:** High-resolution MS data and negative ion MS^2^, MS^3^ and MS^4^ fragmentation of phenolic compounds identified in ‘Novosadska’ buckwheat (*Fagopyrum esculentum*) samples (leaf, stem, flower, and grain)

No	Compound name	*t*_R_, min	Molecular formula, [M − H]^−^	Calculated mass, [M − H]^−^	Exact mass, [M − H]^−^	*Δ*, ppm	MS^2^ fragments, (% base peak)	MS^3^ fragments, (% base peak)	MS^4^ fragments, (% base peak)	Ref.
**Phenolic acids and their derivatives**										
1	Galloyl hexoside isomer 1	1.98	C_13_H_15_O_10_^−^	331.06707	331.06707	0.00	125(9), **169**(100), 170(4)	**125**(100)	79(17), **81**(100), 97(55), 107(13), 163(16)	40
2	Gallic acid^a^	2.39	C_7_H_5_O_5_^−^	169.01425	169.01421	0.24	84(3), 123(8), 124(5), **125**(100), 126(8)	69(49), 79(8), 81(93), 83(53), **97**(100), 107(11)	ND	—
3	Galloyl hexoside isomer 2	3.93	C_13_H_15_O_10_^−^	331.06707	331.06699	0.25	125(5), **169**(100), 170(7), 285(3)	**125**(100)	**69**(100), 80(50)	40
4	Dihydroxybenzoyl hexoside isomer 1	3.96	C_13_H_15_O_9_^−^	315.07216	315.07211	0.16	108(6), 109(10), 152(28), **153**(100), 154(7), 163(6), 165(9)	108(10), **109**(100)	**81**(100)	41
5	Dihydroxybenzoyl hexoside isomer 2	4.42	C_13_H_15_O_9_^−^	315.07216	315.07184	1.00	109(5), **153**(100), 154(4)	**109**(100)	ND	41
6	Protocatechuic acid^a^	4.50	C_7_H_5_O_4_^−^	153.01933	153.01917	1.09	107(3), **109**(100), 110(8), 123(7)	65(42), **81**(100), 91(68), 106(17)	ND	—
7	3-*O*-Caffeoylquinic acid^a^	4.72	C_16_H_17_O_9_^−^	353.08781	353.08614	4.73	135(6), 179(29), **191**(100), 192(4)	**85**(100), 93(62), 109(24), 111(41), 127(95), 173(73)	ND	42
8	Feruloylquinic acid hexoside	5.07	C_23_H_29_O_14_^−^	529.15628	529.15540	1.67	**367**(100), 368(16)	133(8), 135(11), **161**(100), 193(10), 335(6)	**133**(100)	43
9	Caffeoyl hexoside	5.26	C_15_H_17_O_9_^−^	341.08781	341.08775	0.15	135(10), **179**(100), 180(9)	**135**(100)	**107**(100)	41
10	3-*O-p*-Coumaroylquinic acid	5.30	C_16_H_17_O_8_^−^	337.09289	337.09253	1.08	119(6), **163**(100), 164(5), 173(4), 191(7)	**119**(100)	ND	44
11	5-*O*-Caffeoylquinic acid^a^	5.36	C_16_H_17_O_9_^−^	353.08781	353.08780	0.01	179(3), **191**(100)	**85**(100), 93(66), 109(20), 111(37), 127(89), **173**(70)	**57**(100)	—
12	Coumaroyl hexoside	5.39	C_15_H_17_O_8_^−^	325.09289	325.09286	0.10	119(9), **163**(100), 164(5), 289(18)	**119**(100)	ND	45
13	*p*-Hydroxybenzoic acid^a^	5.50	C_7_H_5_O_3_^−^	137.02442	137.02425	1.25	**93**(100), 94(6), 109(3)	ND	ND	—
14	3-*O*-Feruloylquinic acid	5.62	C_17_H_19_O_9_^−^	367.10346	367.10290	1.50	134(6), **193**(100), 194(3)	117(3), **134**(100), 149(26)	**106**(100)	41
15	Caffeic acid^a^	5.89	C_9_H_7_O_4_^−^	179.03498	179.03497	0.05	134(7), **135**(100)	77(5), 78(4), 79(3), 91(56), **107**(100), 117(16)	ND	—
16	5-*O-p*-Coumaroylquinic acid isomer 1	6.01	C_16_H_17_O_8_^−^	337.09289	337.09268	0.64	163(4), **191**(100), 192(3)	**85**(100), 87(22), 93(58), 111(29), 127(95), 173(72)	ND	44

**Phenolic acids and their derivatives**										
17	5-*O-p*-Coumaroylquinic acid isomer 2	6.40	C_16_H_17_O_8_^−^	337.09289	337.09272	0.50	163(3), **191**(100), 192(4)	**85**(100), 93(55), 111(35), 127(95), 171(28), 173(68)	ND	44
18	Methyl 5-*O*-caffeoylquinate isomer 1	6.51	C_17_H_19_O_9_^−^	367.10346	367.10203	3.87	134(5), 135(44), 136(3), 161(11), **179**(100), 180(8), 191(20)	**135**(100)	79(53), **107**(100), 151(18)	31
19	Galloyl-coumaroyl hexoside	6.64	C_22_H_21_O_12_^−^	477.10385	477.10295	1.88	169(9), 287(4), 307(3) **331**(100), 332(12), 433(4)	125(13), **169**(100)	**125**(100)	46
20	*p*-Coumaric acid^a^	6.78	C_9_H_7_O_3_^−^	163.04007	163.03970	2.27	**119**(100), 120(4)	91(5), **93**(100)	ND	—
21	Methyl 5-*O*-caffeoylquinate isomer 2	6.83	C_17_H_19_O_9_^−^	367.10346	367.10257	2.42	134(4), 135(44), 136(3), 161(11), **179**(100), 180(7), 191(19)	**135**(100)	81(36), 93(11), 106(5), **107**(100)	31
22	Methyl-ellagic acid isomer 1	7.06	C_15_H_7_O_8_^−^	315.01464	315.01363	3.22	151(44), 167(26), 175(31), **179**(100), 209(35), 269(60), 300(26)	**151**(100)	ND	47
23	Dicaffeoylquinic acid	7.24	C_25_H_23_O_12_^−^	515.11950	515.11895	1.06	**353**(100), 354(10), 447(5)	135(10), 173(4), 179(46), **191**(100)	85(88), 93(81), 111(49), 127(96), **173**(100)	35
24	Dihydroxybenzoyl-coumaroyl hexoside	7.67	C_22_H_21_O_11_^−^	461.10894	461.10797	2.10	153(3), **315**(100), 316(8)	108(11), 109(11), 152(48), **153**(100), 163(8), 165(13)	108(5), **109**(100)	48
25	Dihydroxybenzoyl-feruloyl hexoside	7.84	C_23_H_23_O_12_^−^	491.11950	491.11908	0.85	153(6), 161(9), **315**(100), 316(11), 323(30), 447(14), 459(22)	108(9), 109(12), 152(38), **153**(100), 163(9), 165(14)	108(9), **109**(100)	48
26	Ferulic acid^a^	8.22	C_10_H_9_O_4_^−^	193.05063	193.05036	1.41	129(4), 133(7), 134(34), **147**(100), 149(5), 161(47), 178(15)	57(7), 85(13), 101(13), 103(22), 111(8), **129**(100)	55(9), 57(60), 73(3), **85**(100)	—
27	Methyl dicaffeoylquinate	8.22	C_26_H_25_O_12_^−^	529.13515	529.13304	3.98	161(5), 179(4), 349(6), **367**(100), 368(9)	133(7), 135(53), 161(78), **179**(100), 191(21), 193(8)	**135**(100)	49
28	Methyl-ellagic acid isomer 2	9.19	C_15_H_7_O_8_^−^	315.01464	315.01434	0.94	179(73), 180(4), 269(16), 271(6), 287(15), **300**(100), 301(18)	243(4), 254(11), 255(54), **271**(100), 272(11)	ND	47

**Flavonoids and their derivatives**										
29	B Type procyanidin dimer isomer 1	5.00	C_30_H_25_O_12_^−^	577.13515	577.13440	1.31	287(8), 289(26), 407(55), **425**(100), 426(9), 451(26), 559(8)	273(7), 381(5), **407**(100)	281(84), 283(35), **285**(100), 297(30), 389(30)	27
30	B Type procyanidin dimer gallate	5.17	C_37_H_29_O_17_^−^	745.14102	745.13843	3.48	315(11), 423(12), 441(38), 467(21), **593**(100), 727(19)	289(8), 315(34), 397(5), 423(17), **441**(100), 467(30)	153(36), 287(17), 289(21), 297(8), **315**(100)	50
31	Catechin^a^	5.43	C_15_H_13_O_6_^−^	289.07176	289.07175	0.05	179(13), 203(10), 205(40), 231(7), **245**(100), 246(11), 247(6)	161(19), 175(9), 187(22), 188(13), **203**(100), 227(25)	157(19), 161(33), **175**(100), 185(20), 188(57)	—
32	Methyl-B type prodelphinidin dimer	5.61	C_30_H_25_O_12_^−^	607.14571	607.14453	1.94	287(45), 405(47), 423(15), 437(58), 449(25), **455**(100), 575(65)	243(30), 303(15), 315(8), **405**(100), 423(27), 437(82)	**243**(100), 283(4)	50
33	B Type procyanidin dimer isomer 2	5.62	C_15_H_13_O_6_^−^	577.13515	577.13409	1.84	287(6), 289(18), 407(47), 408(6), **425**(100), 426(8), 451(20)	273(7), 339(3), 381(5), **407**(100)	281(85), 283(41), **285**(100), 297(33), 389(36)	27
34	Epicatechin^a^	5.93	C_45_H_37_O_18_^−^	289.07176	289.07074	3.53	179(9), 203(7), 205(28), 231(4), **245**(100), 246(6), 247(4)	161(19), 175(9), 187(25), 188(13), **203**(100), 227(26)	161(33), 174(8), **175**(100), 185(21), 188(65)	—
35	B Type procyanidin trimer	5.96	C_21_H_19_O_11_^−^	865.19854	865.19751	1.19	287(26), 407(40), 425(31), 577(59), **595**(100), 713(41), 739(94)	289(24), 405(33), 451(31), 525(37), **543**(100), 677(33)	391(38), 499(8), **525**(100)	51
36	Luteolin 6 *C*-glucoside^a^	6.15	C_37_H_29_O_16_^−^	447.09329	447.09291	0.84	**327**(100), 328(11), 357(33), 358(6), 429(8)	284(6), **299**(100), 300(7)	175(48), 213(66), 240(43), **255**(100), 271(47)	—
37	B Type procyanidin dimer gallate isomer 1	6.18	C_15_H_9_O_8_^−^	729.14611	729.14378	3.19	289(22), **407**(100), 441(38), 451(40), 559(73), 577(63), 603(35)	255(21), 256(19), 283(30), **285**(100), 297(29), 389(20)	213(4), 241(4), **257**(100)	27
38	Myricetin^a^	6.23	C_21_H_19_O_11_^−^	317.03029	317.03015	0.43	163(14), **191**(100), 207(23), 208(4), 255(6), 273(7), 299(31)	135(5), **163**(100)	91(4), 107(24), 119(56), **135**(100)	—
39	Luteolin 8 *C*-glucoside^a^	6.31	C_27_H_29_O_16_^−^	447.09329	447.09177	3.38	**327**(100), 328(6), 357(35), 358(3), 369(5), 393(3)	191(3), 255(3), 284(17), **299**(100), 300(3)	175(41), 199(35), 213(61), 240(43), **255**(100)	—

**Flavonoids and their derivatives**										
40	Quercetin 3-*O*-(6′′-rhamnosyl)-glucoside^a^	6.51	C_27_H_29_O_16_^−^	609.14611	609.14385	3.71	179(3), 255(4), 271(6), 300(31), **301**(100), 302(17), 343(6)	151(75), **179**(100), 255(27), 257(14), 271(47), 272(17)	151(100)	—
41	Apigenin 8 *C*-glucoside^a^	6.65	C_21_H_19_O_10_^−^	431.09837	431.09723	2.64	**311**(100), 312(11), 341(15), 342(3)	**283**(100), 284(3)	163(34), 183(47), 211(29), 224(50), **239**(100)	—
42	Quercetin 3-*O*-glucoside^a^	6.77	C_22_H_17_O_10_^−^	463.08820	463.08774	1.00	300(36), **301**(100), 302(13)	151(79), **179**(100), 255(25), 257(15), 271(34), 273(16)	**151**(100)	—
43	(Epi)catechin gallate^a^	6.84	C_22_H_17_O_10_^−^	441.08272	441.08198	1.67	169(15), 193(5), 271(8), **289**(100), 303(3), 331(11)	179(12), 203(9), 205(34), 231(6), **245**(100), 247(6)	161(19), 187(20), 188(13), **203**(100), 227(30)	—
44	Kaempferol 7-*O*-(6′′-rhamnosyl)-hexoside	6.93	C_27_H_29_O_15_^−^	593.15119	593.14941	3.00	229(3), 257(4), **285**(100), 286(12)	213(27), 229(50), 241(36), 256(21), **257**(100), 267(49)	163(69), 185(20), 213(23), **229**(100), 239(36)	40
45	Quercetin 3-*O*-pentoside	6.99	C_20_H_17_O_11_^−^	433.07764	433.07755	0.20	300(26), **301**(100), 302(8)	151(76), **179**(100), 255(17), 257(12), 271(19), 273(19)	**151**(100)	40
46	Methyl-(epi)gallocatechin gallate	7.03	C_23_H_19_O_11_^−^	471.09329	471.09266	1.34	169(15), **287**(100), 288(10), 303(19), 313(24), 319(34), 439(42)	**125**(100), 161(9), 243(14), 245(3)	**57**(100)	50
47	B Type procyanidin dimer gallate isomer 2	7.19	C_37_H_29_O_16_^−^	729.14611	729.14435	2.42	**407**(100), 408(20), 441(22), 451(21), 559(59), 577(46), 711(33)	255(21), 281(22), 283(33), **285**(100), 297(35), 389(19)	213(5), 241(3), **257**(100)	27
48	Quercetin 3-*O*-rhamnoside^a^	7.27	C_21_H_19_O_11_^−^	447.09329	447.09261	1.51	300(21), **301**(100), 302(8)	151(83), **179**(100), 255(27), 271(34), 272(14), 273(18)	**151**(100)	—
49	Methyl-(epi)catechin gallate	7.41	C_23_H_19_O_10_^−^	455.09837	455.09814	0.50	183(15), 271(6), **289**(100), 290(9), 315(3), 345(3)	179(12), 203(9), 205(36), 231(6), **245**(100), 247(5)	161(18), 187(23), 188(12), **203**(100), 227(29)	27
50	Dimethyl-B type procyanidin dimer gallate	7.49	C_39_H_33_O_16_^−^	757.17741	757.17719	0.29	287(7), 407(9), 559(8), 587(41), **605**(100), 631(24), 739(4)	389(29), 437(37), **587**(100)	389(40), **437**(100)	27

**Flavonoids and their derivatives**										
51	Luteolin 7-*O*-glucoside^a^	7.56	C_21_H_19_O_11_^−^	447.09329	447.09255	1.64	**285**(100), 286(8)	151(35), 175(90), 199(85), 217(71), **241**(100), 243(60)	185(36), 197(96), **198**(100), 199(59), 213(55)	—
52	Kaempferol 3-*O*-rhamnoside	7.78	C_21_H_19_O_10_^−^	431.09837	431.09787	1.17	255(6), 284(49), **285**(100), 286(15), 327(4)	229(30), 241(26), **255**(100), 256(62), 257(80), 267(35)	210(5), 211(59), 212(4), 213(3), **227**(100)	44
53	Quercetin 3-*O*-(6′′-*p*-coumaroyl)-hexoside	8.30	C_30_H_25_O_14_^−^	609.12498	609.12445	0.88	299(3), 300(12), **301**(100), 302(12), 445(12), 463(50), 464(8)	151(90), **179**(100), 229(8), 255(6), 257(21), 271(9)	**151**(100)	44
54	Luteolin^a^	8.79	C_15_H_9_O_6_^−^	285.04046	285.03970	2.68	151(39), 175(92), 199(86), 201(25), 217(78), **241**(100), 243(60)	185(15), **197**(100), 198(78), 199(80), 212(13), 213(58)	152(16), 155(13), **169**(100), 179(15), 182(15)	—
55	Quercetin^a^	8.85	C_15_H_9_O_7_^−^	301.03538	301.03516	0.72	151(82), **179**(100), 180(8), 193(7), 257(12), 271(11), 273(15)	**151**(100)	63(4), 65(3), 83(13), **107**(100)	—
56	Quercetin 3-methyl ether	9.16	C_16_H_11_O_7_^−^	315.05103	315.05066	1.16	**300**(100), 301(10)	243(3), 254(9), 255(52), **271**(100), 272(6)	199(16), 215(18), 227(67), 229(9), **243**(100)	35
57	Kaempferol^a^	9.82	C_15_H_9_O_6_^−^	285.04046	285.04022	0.85	151(71), 185(83), 213(72), **229**(100), 239(78), 255(80), 257(87)	141(15), 145(17), **185**(100), 187(41), 201(93), 211(43)	142(91), **157**(100), 167(16)	—
58	Dimethyl quercetin	10.32	C_17_H_13_O_7_^−^	329.06668	329.06650	0.52	**314**(100), 315(11)	271(3), **299**(100)	227(6), 243(5), 255(9), **271**(100)	35
59	Kaempferide^a^	12.03	C_16_H_11_O_6_^−^	299.05611	299.05597	0.47	165(6), 271(5), **284**(100), 285(10)	**151**(100), 164(24), 227(17), 228(22), 240(15), 255(20)	63(3), 65(3), 83(12), **107**(100), 122(5)	—

a Confirmed using available standards; ND – not detected.

**Fig. 2:**
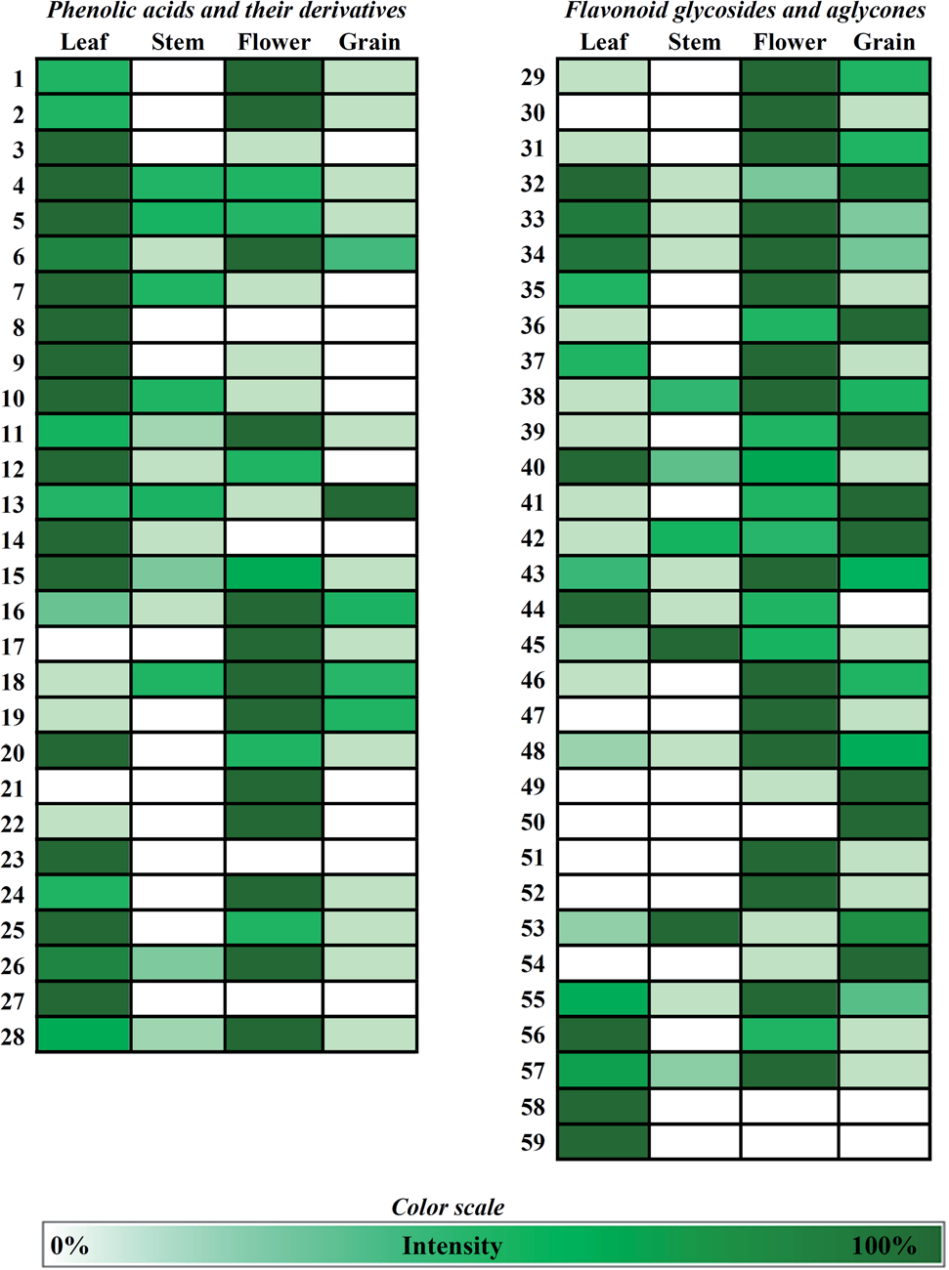
Frequency of phenolic compounds in buckwheat extracts (intensity refers to the obtained peak area of one compound per sample).

The frequency of phenolic compounds in the buckwheat sample extracts is presented as colour scale, which defined the intensity of the obtained peak area of one compound per sample (Fig. 2). The highest number of phenolic compounds was found in the analysed buckwheat flower extract sample (52 phenolic compounds), and then in leaf (50 phenolic compounds) followed by grain (45 phenolic compounds) and stem (27 phenolic compounds) (Fig. 2).

In general, the presence of phenolic acids in analysed buckwheat sample extracts was similar to the results reported by other authors.^23,25,31,39^ Several phenolic acids such as protocatechuic acid (no 6), *p*-hydroxybenzoic acid (no 13) and ferulic acid (no 26), already reported as present in buckwheat,^39^ were also found as common for all buckwheat sample extracts in this study (Fig. 2). The ferulic acid was also found in buckwheat honey from Poland, previously analysed, but not in buckwheat nectar, pollen and Serbian honey sample.^31^ The intensity of the ferulic acid was the most prominent in the buckwheat flower, followed by leaf, and then stem and grain (Fig. 2). Contrary, Dziedzic *et al.*^25^ reported a dominant amount of ferulic acid in the buckwheat roots and that it occurs in the buckwheat stem. Other authors^27^ noted that the buckwheat leaf was the richest in ferulic acid among other compounds. Furthermore, the ferulic acid was found in buckwheat extracts using water, neither using the methanol aqueous extraction solution,^25^ nor using ethanol aqueous solution.^17^ Along with the compounds no 6, 13 and 26, the common phenolic acids for the analysed samples (leaf, stem, flower, grain) were also caffeic acid (compound no 15), derivatives of protocatechuic acid (compounds no 4 and no 5), 5-*O*-caffeoylquinic acid (compound no 11) and its derivative compound no 18, as well as compound no 16, and methyl-ellagic acid isomer 2 (compound no 28) (Fig. 2). In addition to the analysed plant samples, compound no 18 (methyl 5-*O*-caffeoylquinate isomer 1) was also found in buckwheat plant and pollen extract (Fig. 2), but not in nectar or honey.^31^ As we did not found 5-*O-p*-Coumaroylquinic acid in the earlier analysed buckwheat nectar or pollen, but only in buckwheat honey extract,^31^ its appearance, in the form of two isomers, was found in the samples from this study. One isomer (compound no 16) was found in all analysed parts of buckwheat, while another isomer (compound no 17) was present only in buckwheat flower and grain extract (Fig. 2). The compound 3-*O*-caffeoylquinic acid was reported to be present in buckwheat seeds with the highest accumulation in variety ‘Novosadska’.^23^ The presence of this compound (no 7) was confirmed in our analysed buckwheat sample extracts in the following order leaf > stem > flower (Fig. 2). Noticeably, it was not found in buckwheat grain extract (Fig. 2), neither in pollen and in nectar.^31^ The gallic acid was reported to be present in buckwheat sample extracts,^17,24,25,39^ as we noted in this study (Fig. 2). No presence of gallic acid in the buckwheat stem extract (Fig. 2) was also reported by Dziedzic *et al.*^25^

In point of view of presence of flavonoids and derivatives, obtained results were in accordance to many other buckwheat studies.^11,22,29,31,52^ All analysed extracts of green parts of the buckwheat (leaf, stem, flower and grain) showed the presence of quercetin (compound no 55) and its derivatives quercetin 3-*O*-(6′′-rhamnosyl)-glucoside (compound no 40), quercetin 3-*O*-glucoside (compound no 42), quercetin 3*-O*-pentoside (compound no 45), quercetin 3-*O*-rhamnoside (compound no 48) and quercetin 3-*O*-(6′′-*p*-coumaroyl)-hexoside (compound no 53) (Fig. 2). Of eight identified quercetin derivatives, six of them (compound no 40, 42, 45, 48, 53 and 55) were found in all parts of buckwheat plant (Fig. 2). Quercetin 3-methyl ether (compound no 56) was found in the buckwheat extracts of leaf, flower, grain, while derivative dimethyl quercetin (compound no 58) was found only in the buckwheat leaf extract (Fig. 2). Both of these quercetin derivatives were identified in earlier reported corresponding Quercetin and its derivatives are valuable phenolic compounds, with various antioxydative possibilities.^11,17,23,28^ It was noticeable that quercetin, rutin and quercitrin (compound no 55, 40 and 48, respectively) were common polyphenols in the analysed extract of buckwheat samples such as leaf, stem, flower, grain, pollen, nectar, honey from Serbia and Poland (Fig. 2). Accordingly, it was published that quercetin and rutin occur in different aerial parts of buckwheat,^52^ while quercitrin was found only in flowers.^18^ Rutin could be mainly located in the buckwheat leaves,^11^ or according to others, it takes place in the buckwheat flowers.^22^ In this study, rutin (compound no 40) was the most present in the buckwheat leaf, followed by flower, stem and then grain sample (Fig. 2). Quercetin 3-*O*-pentoside (compound no 45) was also confirmed in the buckwheat parts such as leaf, stem and root by other authors.^52^ Quercetin 3-*O*-glucoside (compound no 42) was identified only in the whole buckwheat flour, among other analysed flour samples.^30^

In addition to quercetin derivatives, in the analysed sample extracts were present common six more flavonoids, such as methyl-B type prodelphinidin dimer (compound no 32), B type procyanidin dimer isomer 2 (compound no 33), epicatechin (compound no 34), myricetin (compound no 38), (epi)catechin gallate (compound no 43) and kaempferol (compound no 57) (Fig. 2). Myricetin (compound no 38) was found in all parts of the buckwheat plant (Fig. 2). Many other authors also reported its presence in buckwheat.^29,52^ As a pollen-nectar derived flavonoid^53^ it was also confirmed in buckwheat pollen,^31^ as well as in the buckwheat honey extract.^31,32^ Kaempferide (compound no 59) was found in the analysed buckwheat leaf extract sample (Fig. 2), which is contrary to the results published by Li *et al.*^26^ who did not found it in the leaves from *Fagopyrum esculentum*, but only in the leaves from *Fagopyrum tataricum*. In addition to kaempferide, dimethyl quercetin (compound no 58) was also found in the analysed buckwheat leaf extract sample without appearing in the other samples, while dimethyl-B type procyanidin dimer gallate was identified only in buckwheat grain extract sample (compound no 50) (Fig. 2).

Our observation for the valuable flavan-3-ols, was similar to the already published studies, as this group of compounds were already confirmed to be characteristic for buckwheat.^17,23,24,30^ The identified flavan-3-ols were confirmed by the comparison of their MS fragmentation with the published data.^27,50,51^ All the identified flavan-3-ols (13 of them) were found in the buckwheat grain extract sample (Fig. 2). Flavan-3-ols could express many beneficial effects for human health, as they have better antioxidative effects than rutin shows.^14^ Their presence in the analysed buckwheat sample extracts, primarily in the buckwheat grain, increases the significance of buckwheat. Some of flavan-3-ols were reported to be present in different buckwheat sample extracts such as buckwheat seed^23,39^ grain,^17^ bran,^24^ flour,^14,24,30^ hull,^24^ leaf.^27^ A notable observation for flavanol-3-ols was no occurrence of compounds no 34, no 47, and no 49 in the buckwheat leaf extract (Fig. 2). On the contrary, the buckwheat flower and the grain extract samples have shown more similarity in appearances of the compounds mentioned above and more other flavan-3-ols (Fig. 2). Catechin (compound no 31) was found in the analysed buckwheat extract samples, except in the stem (Fig. 2), which is in accordance with the other authors.^25,52^ Its accumulation was higher in the buckwheat flower, than in the grain and the leaf extract sample (Fig. 2). (Epi)catechin (compound no 34) and some procyanidins B type are significant compounds for buckwheat, as they were not found in cereals such as barley or spelt.^16,17^ In our study, procyanidins had a broader appearance than other identified flavanols (Table 2), and their number may be arranged as follows buckwheat grain > flower > leaf. However, the intensity of these compounds (no 29, 30, 32, 33, 35, 37, and 47) was still higher in the flower and the leaf extract sample (Fig. 2).

Flavone *C*-glycosides were already reported to be present in buckwheat,^26^ as well as in the buckwheat leaf, flower, but not in stem^27^ which was also confirmed in this study (Fig. 2). Luteolin 6-*C*-glucoside, luteolin 8-*C*-glucoside and apigenin 8-*C*-glucoside (compound no 36, 39 and 41, respectively) were found in the analysed buckwheat leaf, flower and grain extracs (Fig. 2). Compound no 41 was reported to be present in buckwheat but not in barley,^17^ as well as in bran obtained from the whole buckwheat, in both free and bound form.^30^ Luteolin is a valuable flavonoid due to its ability to influence on a chronic disease and the most common form of its appearance in plants is as an aglycone or as a glycoside (mostly with glucose).^54^ Dziedzic *et al.*^25^ reported the presence of luteolin in water extract solutions of buckwheat flower and leaf. Contrary to that, we did not identify its presence (compound no 54) in the buckwheat leaf extract, neither in the stem, but it was present in buckwheat flower and grain (Fig. 2) and previously published buckwheat nectar extract sample.^31^ We supposed that the enhanced accumulation of sucrose in the buckwheat leaf extract previously noted (Table 1), and luteolin's propensity to maintain in a bounded form,^54^ could be a reason for it not to appear in the leaf extract. Nonetheless, one more bounded form of luteolin (luteolin 7-*O*-glucoside, compound no 51) was not found in our leaf extract sample (Fig. 2). An explanation could be the possibility that in further fragmentation it can produce luteolin (Table 2). By observing the fragmentation pathway of this compound (no 51), it could be noted that it was the only one whose fragmentation corresponded to the MS data of luteolin. According to these results, luteolin accumulation in the leaf extract was probably excluded in a period of sampling. Further, in the contrast to the published results of the appearing acacetin in the buckwheat leaves,^26^ there was no presence of this polyphenol in the analysed buckwheat sample extracts (Fig. 2), but only in the buckwheat nectar earlier reported.^31^

When comparing the phenolic profile for the buckwheat plant (Table 2, Fig. 2) with the previously published buckwheat pollen, nectar and honey extract samples,^31^ more number of phenolic acids, as well as the flavanols was found in green parts of buckwheat plant and less number of flavonoids. The highlights were on no appearance of flavanonols in the analysed buckwheat plant extract, such as pinobanksin and its derivatives, as well as flavonoids pinocembrin, chrysin, and galangin, known as propolis-derived flavonoids.^53^ On the contrary, their presence was confirmed in the analysed buckwheat nectar and the honey extract.^31^ The importance was on the appearance of some of the well-known polyphenols (*p*-hydroxybenzoic acid, protocatechuic acid, 5-*O*-caffeoylquinic acid, quercetin, rutin, quercitrin and kaempferol) that have been found in all buckwheat sample extracts, starting from the buckwheat plant, its various parts, through the buckwheat pollen and nectar and to the buckwheat honey.^31^ From the obtained results of the identified polyphenols, a good correlation was noted through the plant extract samples analysed in this study, as well as with those of buckwheat pollen and nectar.^31^

By comparing the polyphenolic profile starting from the primary place of photosynthesis to the other parts of the plant, a dominant number of compounds in the leaf and the flower extracts could be noted. Therefore, this may indicate the important activities in these parts of the plant during the flowering period. Primarily, the process of photosynthesis, which could be expected to affect the synthesis of the largest number of polyphenols. Additionally, the process of nectar secretion causes an increase in the number of polyphenols.

Therefore, due to the highest content of sucrose in the leaf extract sample (Table 1), whose metabolism has the main contribution to the formation of polyphenols,^12^ it is expected that most polyphenolic compounds and their derivatives are formed in the leaf. In contrast to the similar number of identified phenolic compounds in buckwheat leaf and flower extract sample (50 and 52, respectively), more other differences between them were noted for the appearing of some phenolic compounds, that mostly belong to the group of cinnamic acid derivatives. Moreover, the intensity of common phenolic acids for the leaf and the flower extract samples goes in favour to the leaf (such as cinnamic acids derivatives, compound no 7, 9, 10, 12, 15, 20 (Fig. 2)).

Contrary to that, the intensity of many flavonoids was found to be higher in flower extract sample with the emphasis on the most of flavonol 3-ols such as compound no 29, 31, 33, 34, 35, 37, 43, 46 (Fig. 2).

In addition to the nectar secretion, buckwheat blooming is successive and has a long flowering time, which affects the abundant presence of phenolic compounds in flowers. This was in accordance the obtained information on the content of phytochemicals and with the reported higher levels of flavonoids and the antioxidant activity of the flowering parts of buckwheat,^22^ for which it was claimed to exhibit health effects.^18^ Furthermore, the transport of phytochemicals from the leaf to other plant parts can be followed by their degradation.

The buckwheat stem was shown to be the poorest in the number of total identified phenolic compounds, due to the low number of flavan-3-ols (four of 13 identified), and no content of flavones (Fig. 2). Most of these compounds that were not found in buckwheat stem were present in all other extract samples. Moreover, these compounds may be precipitated at the bottom of the buckwheat stem, as was previously stated^20^ for anthocyanin's compounds in buckwheat. Particularly, our buckwheat samples were not taken out of the ground, but only by cutting the buckwheat plant. This implied that the lowest part of the buckwheat stem was not taken, where the missing flavanols were probably deposited. The appearance of a small number of phenolic compounds in the extract of stem (Fig. 2), as well as low TPC values, could be due to the formation of the alkaline reactions through the phloem channels where phenolic compounds form aggressive quinones.^12^ These observations also prove stem's functionality in the transport of plant secondary metabolites.

Based on a detailed analysis of the content of polyphenols in the buckwheat sample extracts, the transport of polyphenols from the beginning of photosynthesis to other parts of the plant has been successfully monitored.

The occurrence of polyphenols depends on which part of the plant was analysed. The presence and the development of phenolic compounds influence their formation and stability, as well as the environmental factors or treatments.^10,19^ The presence of procyanidins, among other polyphenols, lead to the health application of different parts of buckwheat, contributing to the initiation of many authors of buckwheat as a functional food.^24,25^ Overall, the obtained results for ‘Novosadska’ buckwheat variety showed the similar polyphenols and prominent antioxidant capacity which were also reported by Kiprovski *et al.*^23^

Following the polyphenolic profile through different parts of buckwheat, it can be used for further investigations of the path and mechanism of the action of polyphenols. Currently, it is still difficult to understand the complete transport of polyphenols through the buckwheat, but it could be seen that there certainly occurs a correlation of buckwheat polyphenols with those in nectar and pollen, and even with buckwheat honey.

## Conclusions

The present study provides the insights into the content of the primary and secondary metabolic compounds in buckwheat plant variety ‘Novosadska’. Sugars, known to be the precursors to the synthesis of polyphenols, have shown to have high amount in the analysed samples, with some differences. The sucrose content was found as noticeable in the leaf extract sample, maltose in pollen and erythritol in the grain extract sample. The nectar extract sample possessed the highest content of trehalose, while the stem extract was noted for the high content of panose and the highest content of fructose. Within this, the occurrence and the abundance of polyphenols in the analysed buckwheat sample extracts showed differences. Nevertheless, antioxidant activity reveal the significance of the each analysed part of buckwheat. It is also worth noting that a good correlation between polyphenol profile of the buckwheat leaf, stem, flower and grain, with those of buckwheat pollen and nectar extract was obtained. Therefore, these results can affect the awareness of the importance of the different parts of buckwheat, as well as buckwheat itself. Since buckwheat is a multi-purpose plant, this type of study contributes to wider use and the buckwheat development as a functional food.

## Author contributions

Conceptualization: M. N., U. G., Ž. T.; formal analysis: M. N., U. G., T. T., N. H., M. S.; investigation: M. N., U. G., T. T., N. N.; methodology: L. I., Ž. T.; resources: N. N., S. B., Ž. T.; supervision: U. G., L. I., Ž. T., S. B.; validation: T. T., U. G., Ž. T.; visualization: M. N., U. G., Ž. T., writing: M. N., U. G., Ž. T.

## Conflicts of interest

There are no conflicts to declare.

## Supplementary Material

RA-011-D1RA04250E-s001

## References

[cit1] Martínez-Villaluenga C., Peñas E., Hernández-Ledesma B. (2020). Food Chem. Toxicol..

[cit2] Popović V., Sikora V., Berenji J., Filipović V., Dolijanović Ž., Ikanović J., Dončić D. (2014). Agric. Econ..

[cit3] Zhu F. (2020). Carbohydr. Polym..

[cit4] Taiz L. and Zeiger E., Plant Physiology, Sinauer Associates, 3rd edn, 2002

[cit5] Shen C., Wang J., Shi X., Kang Y., Xie C., Peng L., Dong C., Shen Q., Xu Y. (2017). Front. Plant Sci..

[cit6] Tovignan T. K., Fonceka D., Ndoyeb I., Cisse N., Luquet D. (2016). Field Crop. Res..

[cit7] Gu H., Man Lu M., Zhang Z., Xu J., Cao W., Miao M. (2018). J. Plant Physiol..

[cit8] Fotirić-Akšić M., Tosti T., Sredojević M., Milivojević J., Meland M., Natić M. (2019). Plants.

[cit9] Živanović B., Milić Komić S., Tosti T., Vidović M., Prokić Lj., Veljović Jovanović S. (2020). Plants.

[cit10] Ma W., Kim J. K., Jia C., Yin F., Kim H. J., Akram W., Hu X., Li X. (2019). Metabolites.

[cit11] Suzuki T., Morishita T., Kim S.-J., Park S.-U., Woo S. H., Noda T., Takigawa S. (2015). Jpn. Agric. Res. Q..

[cit12] Feduraev P., Chupakhina G., Maslennikov P., Tacenko N., Skrypnik Lj. (2019). Antioxidants.

[cit13] Kumar V. , Nagar S. and Sharma P., Carbohydrates in Drug Discovery and Development: Synthesis and Application, Elsevier, 2020

[cit14] Quettier-Deleu C., Gressier B., Vasseur J., Dine T., Brunet C., Luyckx M., Cazin M., Cazin J.-C., Bailleul F., Trotin F. (2000). J. Ethnopharmacol..

[cit15] Holasova M., Fiedlerova V., Smrcinova H., Orsak M., Lachman J., Vavreinova S. (2002). Food Res. Int..

[cit16] Bittner K., Rzeppa S., Humpf H. U. (2013). J. Agric. Food Chem..

[cit17] Aleksenko S. S. (2013). Anal. Chem..

[cit18] Zielińska D., Turemko M., Kwiatkowski J., Zieliński H. (2012). Molecules.

[cit19] Kalinova J., Triska J., Vrchotova N. (2006). J. Agric. Food Chem..

[cit20] Huda M. N., Lu S., Jahan T., Ding M., Jha R., Zhang K., Zhang W., Georgiev M. I., Park S. U., Zhou M. (2021). Food Chem..

[cit21] Zhang Z.-L., Zhou M.-L., Tang Y., Li F.-L., Tang Y.-X., Shao J.-R., Xue W.-T., Wu Y.-M. (2012). Food Res. Int..

[cit22] Stojilkovski K., Kočevar Glavač N., Kreft S., Kreft I. (2013). J. Food Compost. Anal..

[cit23] Kiprovski B. B., Mikulic-Petkovsek M., Slatnar A., Veberic R., Stampar F., Malencic Dj., Latkovic D. (2015). Food Chem..

[cit24] Zhang W., Zhu Y., Liu Q., Bao J., Liu Q. (2017). J. Funct. Foods.

[cit25] Dziedzic K., Danuta Górecka D., Szwengiel A., Sulewska H., Kreft I., Gujska E., Walkowiak J. (2018). Plant Foods Hum. Nutr..

[cit26] Li J., Yang P., Yan Q., Gong X., Ma H., Dang K., Chen G., Gao X., Feng B. (2019). Molecules.

[cit27] Dziadek K., Kopeć A., Piątkowska E., Leszczyńska T., Pisulewska E., Witkowicz R., Bystrowska B., Francik R. (2018). Eur. Food Res. Technol..

[cit28] Zhang X.-Y., Chen J., Li X.-L., Yi K., Ye Y., Liu G., Wang S.-F., Hu H.-L., Zou L., Wang Z.-G. (2017). J. Funct. Foods.

[cit29] Kraujalienė V., Pukalskas A., Venskutonis P. R. (2017). Ind. Crops Prod..

[cit30] Inglett G. E., Chen D., Berhow M., Lee S. (2011). Food Chem..

[cit31] Nešović M., Gašić U., Tosti T., Horvacki N., Šikoparija B., Nedić N., Blagojević S., Ignjatović L. J., Tešić Ž. (2020). R. Soc. Open Sci..

[cit32] Kečkeš S., Gašić U., Ćirković-Veličković T., Milojković-Opsenica D., Natić M., Tešić Ž. (2013). Food Chem..

[cit33] Różańska D., Mikoś K., Regulska-Ilow B. (2020). Rocz. Panstw. Zakl. Hig..

[cit34] Kalaycıoglu Z., Kaygusuz H., Doker S., Kolayli S., Bedia Erim F. (2017). LWT - Food Sci. Technol..

[cit35] Gašić U., Kečkeš S., Dabić D., Trifković J., Milojković-Opsenica D., Natić M., Tešić Ž. (2014). Food Chem..

[cit36] Li X., Kim J. K., Park S.-Y., Zhao S., Kim Y. B., Lee S., Park S. U. (2014). J. Agric. Food Chem..

[cit37] Sengupta S., Mukherjee S., Basa P., Majumder A. L. (2015). Front.
Plant Sci..

[cit38] Singh A., Bajpai V., Srivastava M., Ram Arya K., Kumar B. (2015). J. Pharm. Anal..

[cit39] Guo X.-D., Ma Y.-J., Parry J., Gao J.-M., Yu L.-L., Wang M. (2011). Molecules.

[cit40] Aničić N., Patelou E., Papanikolaou A., Kanioura A., Valdesturli C., Arapitsas P., Skorić M., Dragićević M., Gašić U., Koukounaras A., Kostas S., Sarrou E., Martens S., Mišić D., Kanellis A A. (2021a). Front. Plant Sci..

[cit41] Aničić N., Gašić U., Lu F., Ćirić A., Ivanov M., Jevtić B., Dimitrijević M., Anđelković B., Skorić M., Nestorović Živković J., Mao Y., Liu J., Tang C., Soković M., Ye Y., Mišić D. (2021b). Pharmaceuticals.

[cit42] Stojković D., Gašić U., Drakulić D., Zengin G., Stevanović M., Rajčević N., Soković M. (2021). J. Pharm. Biomed. Anal..

[cit43] Jaiswal R., Müller H., Müller A., Karar M. G. E., Kuhnert N. (2014). Phytochemistry.

[cit44] Zengin G., Cvetanović A., Gašić U., Stupar A., Bulut G., Senkardes I., Dogan A., Seebaluck-Sandoram R., Rengasamyg K. R. R., Sinan K. I., Mahomoodally M. F. (2019). Ind. Crop. Prod..

[cit45] Zengin G., Cvetanović A., Gašić U., Dragićević M., Stupar A., Uysal A., Şenkardes I., Sinan K. I., Picot-Allain M. C. N., Ak G., Mahomoodally M. F. (2020). Ind. Crop. Prod..

[cit46] Wang J., Qin Y., Kong W., Wang Z., Zeng L., Fang F., Jin C., Zhao Y., Xiao X. (2012). Food Chem..

[cit47] Zhang H., Yu M., Jia H., Zhang T., Shang H., Zhang M., Zhu Z., Zou Z. (2020). J. Sep. Sci..

[cit48] Aita S. E., Capriotti A. L., Cavaliere C., Cerrato A., Giannelli Moneta B., Montone C. M., Piovesana S., Laganà A. (2021). Separations.

[cit49] Jaiswal R., Kuhnert N. (2011). J. Mass Spectrom..

[cit50] Dai W., Qi D., Yang T., Lv H., Guo L., Zhang Y., Zhu Y., Peng Q., Xie D., Tan J., Lin Z. (2015). J. Agric. Food Chem..

[cit51] Milinčić D., Stanisavljević N., Kostić A., Soković Bajić S., Kojić M., Gašić U., Barać M., Stanojević S., Tešić Ž., Pešić M. (2021). LWT--Food Sci. Technol..

[cit52] Kalinova J., Vrchotova N. (2009). J. Agric. Food Chem..

[cit53] Tomás-Barberán F. A., Ferreres F., Carcia-Viguera C., Tomás-Lorente F. (1993). Z. Lebensm.-Unters.-Forsch..

[cit54] Lopez-Lazaro M. (2009). Med. Chem..

