# Supplementary material for: Distribution of polyphenolic and sugar compounds in different buckwheat plant parts
Source: RSC Adv. 2021 Jul 28;11(42):25816–29. doi: 10.1039/d1ra04250e (PMC9037080; doi:10.1039/d1ra04250e)
Supplement: RA-011-D1RA04250E-s001 [file RA-011-D1RA04250E-s001.pdf]

Electronic Supplementary Material (ESI) for RSC Advances.
This journal is © The Royal Society of Chemistry 2021

Supplementary material

Distribution of polyphenolic and sugar compounds in different buckwheat plant parts

Milica Nešović, ‡[a] Uroš Gašić, ‡*[b] Tomislav Tosti,[c] Nikola Horvacki,[d] Nebojša Nedić,[e] Milica Sredojević,[c] Stevan Blagojević,[a] Ljubiša Ignjatović[f] and Živoslav Tešić*[c]

[a]Institute of General and Physical Chemistry, Studentski trg 12-16, 11158 Belgrade, Serbia

[b]Institute for Biological Research "Siniša Stanković", University of Belgrade, Bulevar despota Stefana 142, 11060 Belgrade, Serbia

[c]University of Belgrade - Faculty of Chemistry, Studentski trg 12-16, 11158 Belgrade, Serbia

[d]University of Belgrade - Faculty of Physical Chemistry, Studentski trg 12-16, 11158 Belgrade, Serbia

[a.]Institute of General and Physical Chemistry, Studentski trg 12-16, 11158 Belgrade, Serbia

[b.]Department of Plant Physiology, Institute for Biological Research "Siniša Stanković", National Institute of Republic of Serbia, University of Belgrade, Bulevar despota Stefana 142, 11060 Belgrade, Serbia

[c.]University of Belgrade - Faculty of Chemistry, Studentski trg 12-16, 11158 Belgrade, Serbia

[d.]Innovation Center, University of Belgrade – Faculty of Chemistry, Studentski trg 12-16, 11158 Belgrade, Serbia

[e.]Faculty of Agriculture, Institute for zootehnics, University of Belgrade, Nemanjina 6, 11080 Belgrade - Zemun, Serbia

[f.]University of Belgrade - Faculty of Physical Chemistry, Studentski trg 12-16, 11158 Belgrade, Serbia

‡ M. Nešović and U. Gašić contributed equally to this work.

*Corresponding author

Table S1

The list of analyzed sugars with validation parameters for quantification.

Sugar compound	R^2	Recovery (%)	CV (%)	LOD (ppm)	LOQ (ppm)
**Glucose**	0.9998	93.58 – 108.22	3,25	2.55	8.52
**Fructose**	0.9988	96.14 – 102.98	6.12	1.46	4.87
**Xylose**	0.9993	98.33 – 104.25	1.58	0.47	1.55
**Arabinose**	0.9963	96.32 – 107.36	1.03	0.33	1.01
**Rhamnose**	0.9991	94.39 – 109.32	4.18	063	2.09
**Sucrose**	0.9990	93.03 – 105.05	4.97	0.19	0.62
**Maltose**	0.9965	93.81 – 103.60	3.35	0.27	0.91
**Isomaltose**	0.9953	95.89 – 105.21	2.98	0.35	1.16
**Trehalose**	0.9989	96.98 – 106.03	2.39	0.40	1.33
**Turanose**	0.9992	91.97 – 105.83	2.75	0.24	0.81
**Melibiose**	0.9974	93.65 – 107.77	1.98	0.35	1.15
**Gentiobiose**	0.9956	92.78 – 105.63	3.68	0.55	1.81
**Melesitose**	0.9957	93.12 – 108.54	3.21	0.27	0.90
**Raffinose**	0.9994	95.63 – 108.36	3.65	0.32	1.06
**Maltotriose**	0.9920	95.36 – 107.32	1.37	0.30	0.99
**Panose**	0.9989	92.31 – 104.63	4.98	0.53	1.75
**Erythritol**	0.9975	90.99 – 104.63	3.65	0.45	1.48
**Glycerol**	0.9974	91.65 – 107.36	2.53	0.19	0.63
**Sorbitol**	0.9994	94.58 – 108.54	1.56	0.22	0.74
**Galactitol**	0.9989	95.87 – 106.32	1.47	0.49	1.62
**Mannitol**	0.9986	92.11 – 106.89	1.95	0.38	1.25

The calibration range was from 10-100 ppm for glucose and fructose, whereas all the others analytes were in the range from 1-10 ppm. The accuracy and precision was confirmed by participation in proficiency testing where all the y scores were lower than 1.

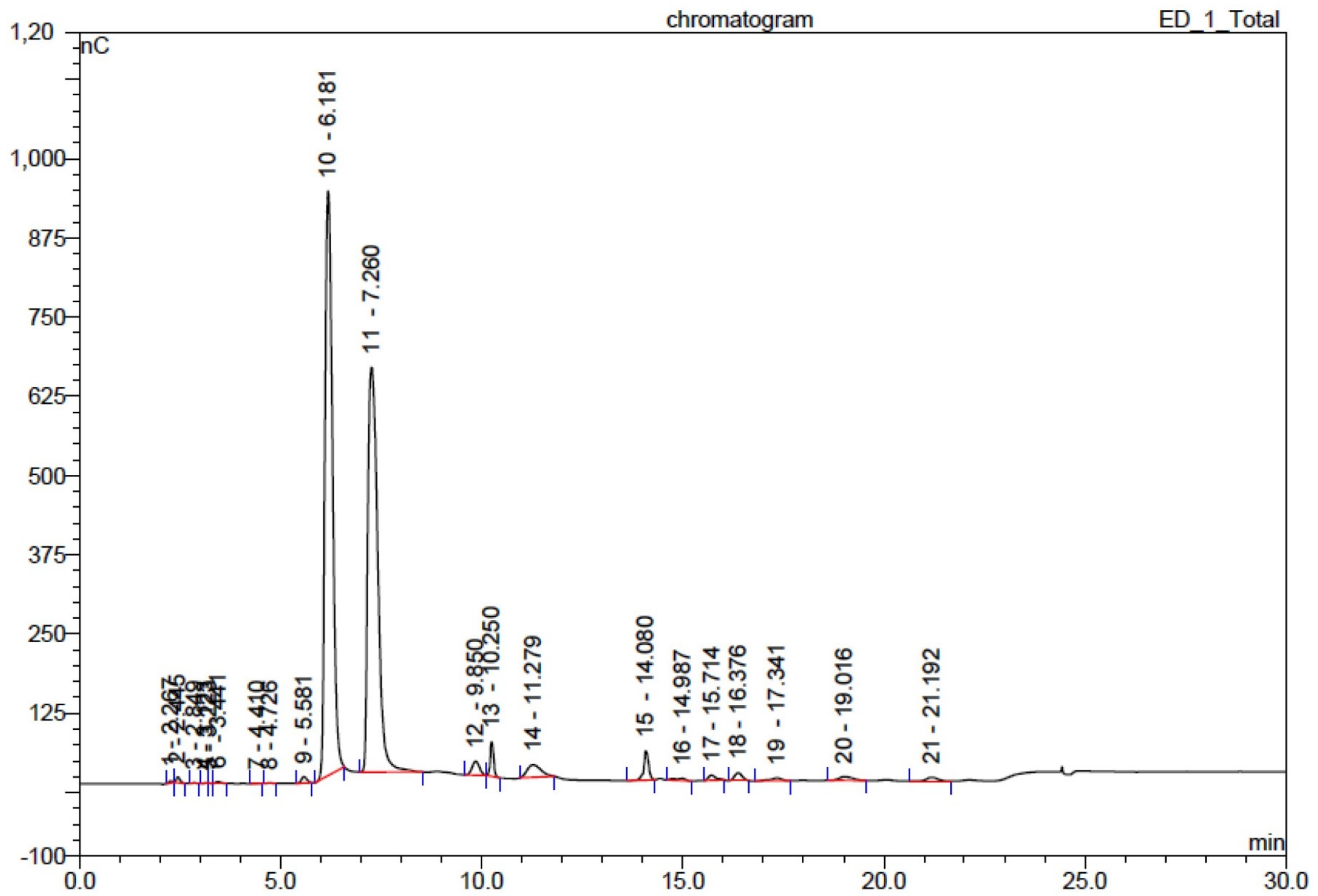

Figure S1. Chromatogram of buckwheat flower extract: 1.Erythritol; 2.Glycerol; 3.Sorbitol; 4.Galactitol; 5. Mannitol; 6.Trehalose; 7. Rhamnose; 8. Arabinose; 9. Xylose; 10. Glucose; 11.Fructose; 12.Melibiose; 13. Isomaltose; 14. Sucrose; 15. Melesitose; 16. Gentiobiose; 17. Turanose; 18. Raffinose; 19. Maltose; 20. Panose; 21. Maltotriose.

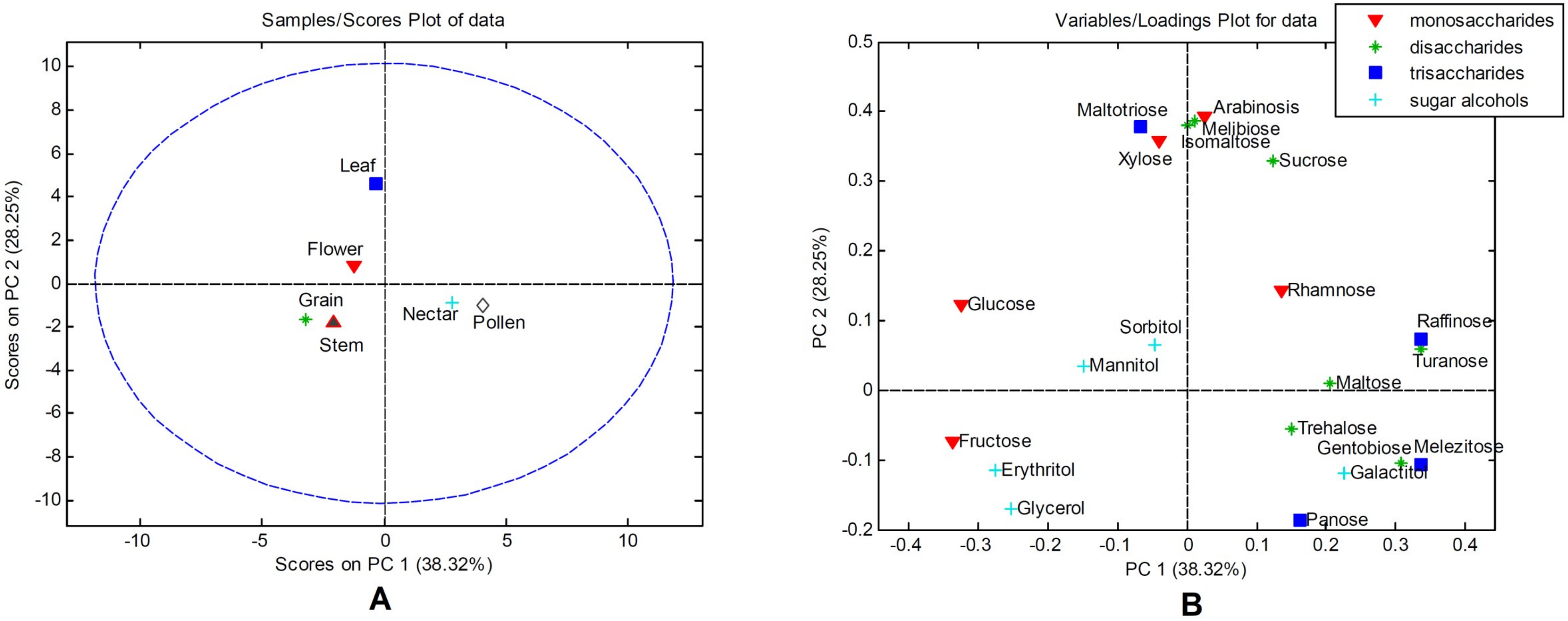

Figure S2. Sugar-based differentiation of buckwheat (*Fagopyrum esculentum*) extract samples. Principal component scores (A) and loadings plot (B).

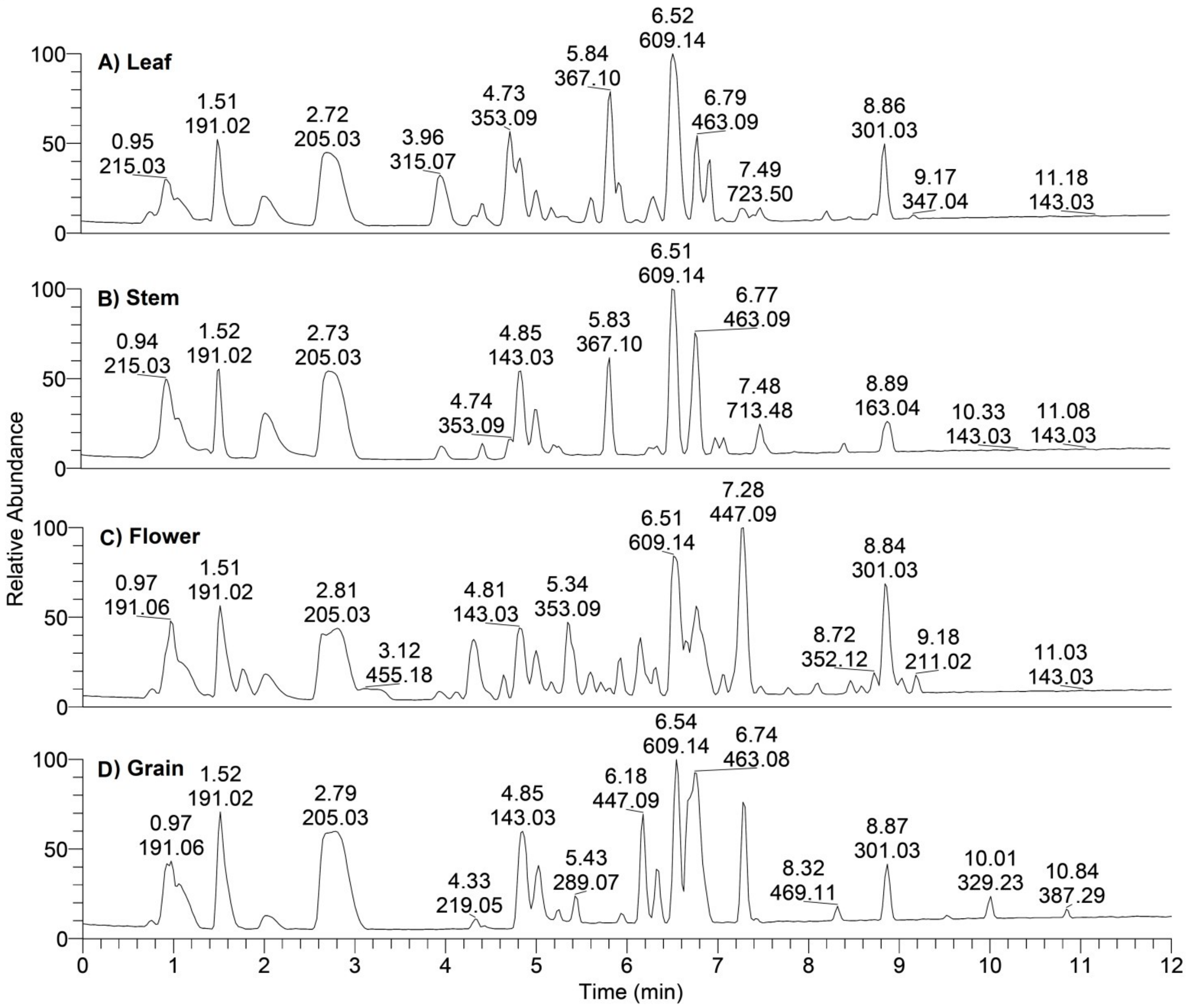

Figure S3. Base peak chromatograms of identified phenolic compounds in the investigated buckwheat sample extracts (the retention times and exact masses from for all peak are listed in the Table 2, and in this picture only the most intense).
